# Waist Circumference Is an Essential Factor in Predicting Insulin Resistance and Early Detection of Metabolic Syndrome in Adults

**DOI:** 10.3390/nu15020257

**Published:** 2023-01-04

**Authors:** José Ignacio Ramírez-Manent, Andrés Martínez Jover, Caroline Silveira Martinez, Pilar Tomás-Gil, Pau Martí-Lliteras, Ángel Arturo López-González

**Affiliations:** 1 Balearic Islands Health Service, 07010 Palma, Balearic Islands, Spain; 2 Faculty of Medicine, University Balearic Islands, 07010 Palma, Balearic Islands, Spain; 3 Institut d’Investigació Sanitària de les Illes Balears (IDISBA), Balearic Islands Health Research Institute Foundation, 07010 Palma, Balearic Islands, Spain; 4 Faculty of Medicine, University School ADEMA, 07010 Palma, Balearic Islands, Spain; 5 Investigation Group ADEMA, SALUD of Instituto Universitario de Investigación en Ciencias de la Salud (IUNICS), 07010 Palma, Balearic Islands, Spain; 6 William Harvey Research Institute, London School of Medicine and Dentistry, Queen Mary University of London, London EC1M 6BQ, UK

**Keywords:** metabolic syndrome, insulin resistance, waist circumference

## Abstract

Background: Metabolic syndrome (Met-S) is considered one of the most important health problems of the 21st century. It includes a group of metabolic disorders that increase the risk of cardiovascular diseases such as overweight and obesity, elevated lipid profile and blood pressure and insulin resistance (IR). Based on the information mentioned above in which there seems to be a relationship between IR and Met-S, the objective of this work was twofold: on the one hand, to assess the relationship between the values of different insulin resistance risk scales and Met-S determined with three different scales, and on the other, to determine whether any of the components of Met-S predispose more to the appearance of IR. Methods: A descriptive cross-sectional study of 418,343 workers. Waist circumference was measured and evaluated together with six formulas to assess the insulin resistance index. Categorical variables were evaluated by calculating the frequency and distribution of each one. For quantitative variables, mean and standard deviation were determined, and Student’s t-test was applied, while for qualitative variables, the chi-square test was performed. The usefulness of the different risk scales for insulin resistance for predicting metabolic syndrome was evaluated using ROC curves, the area under the curve (AUC), as well as their cut-off points for sensitivity, specificity, and the Youden index. Results: People with metabolic syndrome applying any criteria had higher values in the IR risk scales. The different IR scales made it possible to adequately classify people with metabolic syndrome. Of the three definitions of Met-S, the one that showed the greatest relationship with IR was IDF. Conclusions: Most risk scales for insulin resistance enable the presence of metabolic syndrome to be adequately classified, finding the best ones if the International Diabetes Federation (IDF) criteria are applied. Of the elements included in the Met-S, the one that seems to increase the risk of presenting IR the most is waist circumference; hence, the Met-S definition that is most related to IR is that of the IDF, which is the only one of the three in which a high value of waist circumference is necessary to be able to diagnose Met-S. Waist circumference can be considered the central essential component for detecting insulin resistance and, therefore, the early detection of metabolic syndrome.

## 1. Introduction

Metabolic syndrome (Met-S) is considered one of the most important health problems of the 21st century. It includes a group of metabolic disorders that increase the risk of suffering from cardiovascular diseases such as overweight and obesity, elevated lipid profile and blood pressure and insulin resistance [1,2]. Met-S multiplies the risk of type 2 diabetes by five [2,3] and that of other cardiovascular diseases by three [2,4]. Similarly, people with Met-S also have a greater predisposition for polycystic ovaries in women [5]; nonalcoholic fatty liver, especially in men [6]; asthmatic symptoms [7]; sleep problems [8]; certain oncological processes [9,10]; and, in recent years, its relationship with sarcopenia has been evidenced [10].

The clinical entities that constitute Met-S vary according to the criteria that are applied, but in general, they include high blood pressure, high blood glucose values, high waist circumference values, low HDL and high triglyceride levels and insulin resistance [10,11]. The sarcopenia that occurs in Met-S is closely related to the persistent inflammation that arises from this process. People with a greater amount of visceral fat produce a lot of inflammatory cytokines, so the muscle tissue of Met-S patients is in a state of constant inflammation, which increases the risk of muscle atrophy [10].

The origin of insulin resistance (IR) is found in the current Western diet, which, in general, has little nutritional value since it contains a large amount of so-called empty calories that come from the consumption of sugars and refined carbohydrates that have a high glycaemic load [12,13]. This dietary model causes sudden increases in blood glucose that lead to a high insulin response to try to control blood glucose levels, thus causing a drop in blood glucose below normal values that is accompanied by fatigue and, again, from hunger [14]. Prolonged maintenance of this situation causes a greater amount of insulin to be required in order to maintain normal blood glucose levels, a situation that will facilitate the appearance of IR [15].

In IR, although insulinemia is normal, the cells are not able to “translate” the insulin signals, so the so-called glucose receptor (GluT4) does not move correctly from inside the cell to the membrane, preventing the entry of glucose into it and, therefore, increasing its presence in the blood [16,17]. Our body reacts by increasing insulinemia to try to control hyperglycaemia [17,18].

Excess glucose resulting from IR can accumulate in adipose tissue and in certain viscera. Fat tissue, in this situation, behaves as an endocrine organ and releases inflammatory cytokines (adipocytokines) that lead to a blockade of the insulin signal, which will aggravate IR and cause a certain degree of tissue inflammation [19,20]. However, not only is there an increase in fatty tissue due to the deposition of glucose in the adipocytes, but also the previously mentioned blockade of beta oxidation caused by hyperinsulinemia, which blocks lipolysis [21]. Both processes favour abdominal obesity [22], the formation of triglycerides in the liver [23] and the release of very-low-density lipoproteins (VLDLs), the latter situations of which cause dyslipidaemia [24]. Insulin resistance also facilitates the high blood pressure that occurs in Met-S by losing the vasodilator effect of insulin and the vasoconstriction produced by free fatty acids that produce reactive species and eliminate nitric oxide [25]. All this makes it necessary to find simple and safe indicators that enable the diagnosis of patients with Met-S in an easy way in clinical practice.

Based on the information mentioned above, in which there seems to be a relationship between IR and Met-S, the objective of this work was twofold: on the one hand, to assess the relationship between the values of different insulin resistance risk scales and Met-S determined with three different scales, and on the other, to determine which component of Met-S can be utilised to predict insulin resistance.

## 2. Methods

Cross-sectional study of 418,343 workers (172,282 women and 246,061 men) belonging to different autonomous communities in Spain (Balearic Islands, Andalusia, Canary Islands, Valencian Community, Catalonia, Madrid, Castilla La Mancha, Castilla León, Basque Country) and from different employment sectors, especially hospitality, construction, trade, health, public administration, transport, education, industry and cleaning. The study was carried out between January 2019 and June 2020. The workers were selected from those who underwent periodic occupational medical examinations.

The population for this study was obtained from the anonymised database of workers that is deposited in the repository of the ADEMA-UIB University School (University of the Balearic Islands). This database comes from occupational medical examinations carried out in the last 5 years in various occupational risk prevention services throughout Spain (RD 688/2005 as of 10 June and Law 31/95 on occupational risk prevention). Anonymisation of this database prevents researchers from knowing the identity of the workers.

### 2.1. Inclusion Criteria

- Working age (18 and 67 years);
- Being an active worker;
- Agreeing to participate in the study.

**Figure 1:**
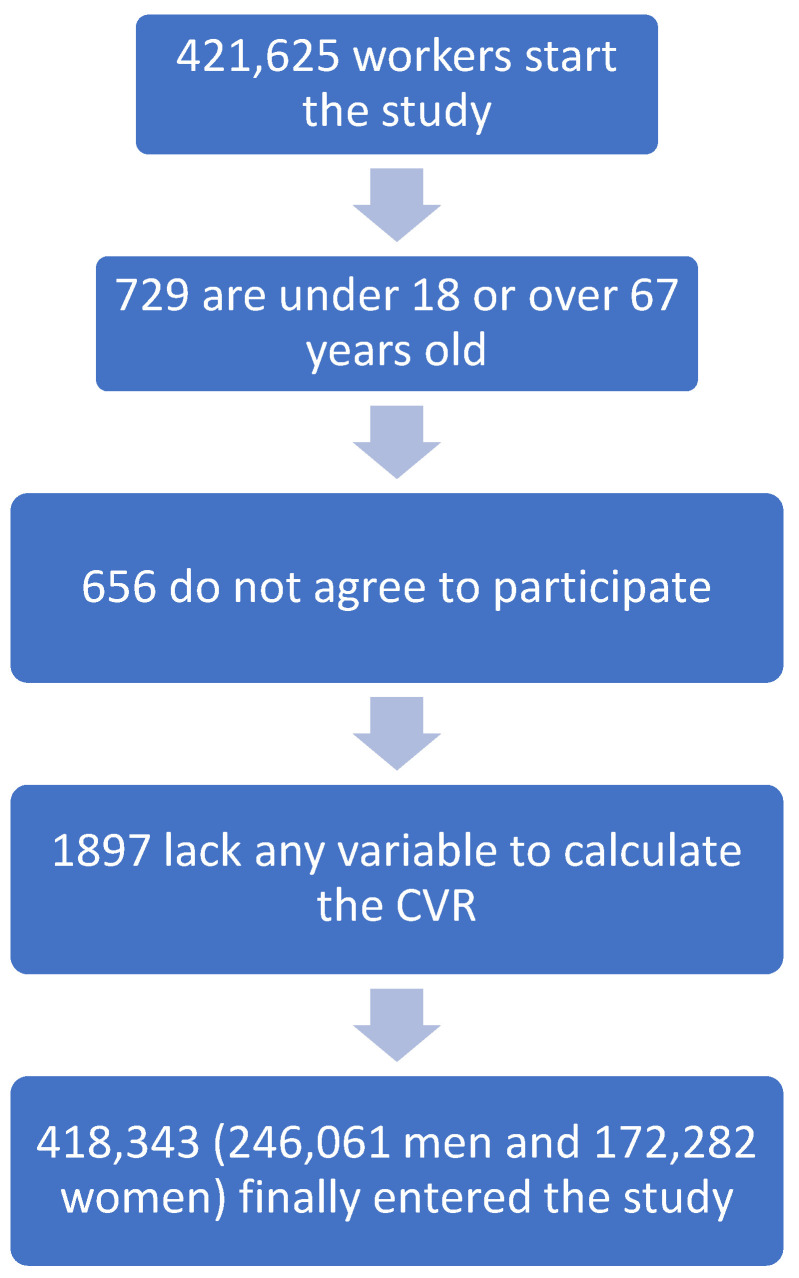
Flowchart.

With these inclusion criteria, the following flowchart was established (Figure 1).

### 2.2. Determination of Variables

The health personnel of the different participating companies, applying standardised protocols, oversaw the obtention of the anthropometric variables (height, weight and waist circumference) and clinical and analytical variables.

Waist circumference, expressed in cm, was measured with a metric tape: SECA model 200 (range 1–200 cm, with millimetre divisions). The worker stood with feet together, trunk straight, abdomen relaxed and arms hanging. In this position, the tape was placed parallel to the ground at the level of the last floating rib. A calibrated OMRON M3 model automatic sphygmomanometer was used to measure blood pressure. With the person seated and after resting for 10 min, three measurements were made at one-minute intervals, and the average was obtained. Blood samples were obtained from peripheral blood after at least 12 h of fasting. They were processed in the different laboratories within a maximum period of 2–3 days. Automated enzymatic techniques were used to determine blood glucose, total cholesterol and triglycerides. HDL cholesterol was obtained by precipitation with dextran-sulphate Cl2Mg. LDL cholesterol was calculated using the Friedewald formula (valid only when triglycerides were less than 400 mg/dL).

Friedewald formula: LDL-c = total cholesterol -HDL-c- triglycerides/5.

All these parameters are expressed in mg/dL.

A person was considered a smoker when they had consumed at least one cigarette each day (or the equivalent in other forms of consumption) in the last month or if they had stopped smoking less than a year before. A person was considered a nonsmoker when they had not used tobacco in the previous 12 months or if they had never smoked.

Social class was classified into three categories and was obtained from the data of the National Classification of Occupations 2011 (CNO-11) applying the criteria of the Spanish Society of Epidemiology [26]: class I includes managerial positions, university professionals, athletes and artists; class II includes skilled workers in intermediate occupations and self-employed workers; and class III includes unskilled workers.

Metabolic syndrome was determined by applying three different criteria [27]: the National Cholesterol Education Program Adult Treatment Panel III (NCEP/ATP-III), the Joint Interim Statement (JIS) and the International Diabetes Federation (IDF) update.

The following risk scales for insulin resistance are calculated:

- Triglycerides/HDL-c. It is obtained by dividing the value of triglycerides by the value of HDL cholesterol. Values over 2.4 [28] are considered high.
- Glucose triglyceride index (TyG index). It is obtained by the formula: Ln(triglycerides [mg/dL] × glucose [mg/dL]/2). High values are considered from 8.8 [28].
- TyG index-BMI [29]. This is obtained by multiplying the TyG index by body mass (BMI).
- TyG index-waist circumference [29]. This is obtained by multiplying the TyG index by waist circumference.
- TyG index-WtHR [30]. This is obtained by multiplying the TyG index by the waist/height index.
- Metabolic score for insulin resistance (METS-IR). Obtained by applying the formula: Ln((2 × G0) + TG0) × BMI)/(Ln(HDL-c)) (G0, fasting glucose; TG0, fasting triglycerides; BMI, body mass index; HDL-c, high-density lipoprotein cholesterol). High values are considered from 50 [31].

### 2.3. Ethical Considerations and Aspects

All procedures were performed applying the ethical standards of the institutional research committee and the 2013 Declaration of Helsinki, paying special attention to the anonymity of the participants and the confidentiality of the data collected. The Research Ethics Committee of the Balearic Islands (CEI-IB) approved the study, which was obtained with the following indicator—IB 4383/20. All participants signed written informed consent documents. The data collected for the study were identified by a code, and only the person responsible for the study could relate these data to the participants. The identity of the participants was not disclosed in any report of this study. The research team undertook to strictly comply with Organic Law 3/2018, as of December 5 in Spain, on the protection of personal data and the guarantee of digital rights, guaranteeing the participants in this study that they could exercise their rights of access, rectification, cancellation and opposition of the data collected.

### 2.4. Statistical Analysis

A descriptive analysis of the categorical variables was performed by calculating the frequency and distribution of each one. For quantitative variables, mean and standard deviation were determined, and the Student’s *t*-test was applied, while for qualitative variables, the chi-square test was performed. To evaluate the usefulness of the different risk scales for insulin resistance for predicting metabolic syndrome, ROC curves were performed, and the area under the curve (AUC) was determined, as well as their cut-off points for sensitivity, specificity, and the Youden index. A multivariate analysis was performed using binary logistic regression. Statistical analysis was performed with the SPSS 28.0 programme, with the accepted level of statistical significance being *p* < 0.05.

## 3. Results

Most of our population were men (58.8%). Most workers were between 30 and 49 years old and belonged to social class III, while 33.2% were smokers. The values of all the anthropometric, clinical and laboratory parameters were higher in men. All data can be found in Table 1.

Table 2 shows that the mean values of all the risk scales for insulin resistance were much higher in people with metabolic syndrome when applying any of the criteria; this situation is observed in both men and women. In all cases, the differences observed are statistically significant.

Table 3 shows the results of the binary logistic regression that reveal how the risk of presenting metabolic syndrome when applying any of the three criteria was much higher in workers who had a high risk of insulin resistance compared with those who had a lower risk. OR values are particularly high when the METS-IR scale was applied.

Table 4 shows how the components of metabolic syndrome, applying the three criteria, increase the risk of presenting high values on the three insulin resistance scales. Of all the components of metabolic syndrome, the one that showed the highest OR value was waist circumference.

**Figure 2:**
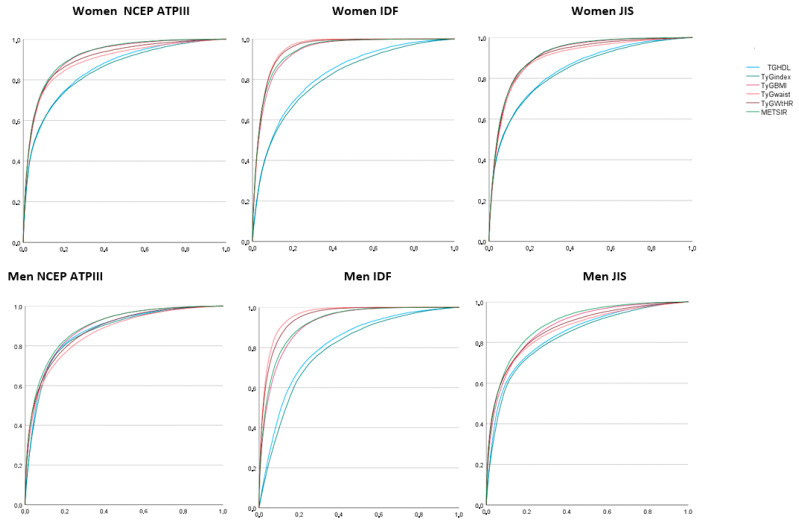
Areas under the curve (ROC curve) of the different insulin resistance risk scales for predicting the presence of metabolic syndrome when applying different criteria.

Figure 2 and Table 5 show the results of the ROC curves, noting that the areas under the curve, in both women and men, are very large for most risk scales for insulin resistance, obtaining the following values: worse results for triglycerides/HDL and the TyG index. The largest areas under the curve were obtained when the IDF criteria for metabolic syndrome were applied and when we referred to the IR scales that included waist circumference in their calculation, that is, the TyG index-BMI and the TyG index-waist/height.

Table 6 shows the cut-off points of the different risk scales for insulin resistance for predicting the presence of metabolic syndrome with the different criteria, as well as the sensitivity, specificity and Youden index of these cut-off points. The best results were obtained when the IDF criteria were applied. Of the IR scales, those with the highest Youden indices—and, therefore, those that combined greater sensitivity and specificity—were those scales in which waist circumference was included; that is, the TyG index-waist and the TyG index-waist/height.

## 4. Discussion

In our study, people with metabolic syndrome, when applying any criteria, presented higher values on the IR risk scales. The different IR scales made it possible to adequately classify people with metabolic syndrome. Of the three definitions of Met-S, the one that showed the greatest relationship with IR was IDF, perhaps because it is the only one that requires the presence of high values of waist circumference for its diagnosis. Previous studies have shown that visceral abdominal fat is the most closely related factor to insulin resistance and that waist circumference is the best marker of metabolic risk of visceral fat [32,33]. A recent study carried out by Deusdará et al. on Brazilian adolescents also found a close relationship between the waist circumference of adolescents and insulin resistance. What they highlight can be very useful in primary care for identifying insulin resistance early on [34].

Like us, different authors have found a relationship between MS and IR. That is, when insulin resistance develops in adipose tissue, inhibition of insulin-regulated lipolysis is avoided, which produces an increase in circulating free fatty acids that increase insulin resistance by producing alterations in insulin signage in different organs, which establishes a vicious circle [35]. As the metabolism of fats is intimately linked, the elevation of free fatty acids in the blood and their metabolism are, in turn, the origin and result of insulin resistance and DM2, producing chronic inflammation, atherosclerosis, destruction of the β cells of the pancreas and other previously described pathologies [36]. This chronic inflammation has been accepted as the main underlying cause of insulin resistance [37], and it can be detected as early as children and teens in which Met-S has been associated with inflammation, insulin resistance, obesity and a sedentary lifestyle [38]. A review by Gluvic et al. [39] in 2017 indicated that people with Met-S had greater cardiometabolic complications, among which we could highlight IR. Other authors such as Brown et al. [40] have also established a close relationship between Met-S and IR, considering that there are genetic aspects that relate them. Some studies have found a significant relationship between the CC genotype of IL-6 −174 G > C and Met-S. Identifying these genetic variants could help identify people at higher risk for Met-S and IR [41]. Recent studies confirm the above, establishing a relationship between insulin resistance, age, the inflammatory state, different diseases and death. Noting that all of this is closely related to obesity [42,43], insulin resistance can be used alone to predict the development of cardiovascular diseases [44,45].

Different studies have addressed the relationship between TyG index and Met-S values, obtaining results similar to ours. The TyG index is a reliable diagnostic indicator of insulin resistance, which is calculated using fasting glucose and TG levels, making it very easy to use using simple routine biochemical tests [46,47]. A study by Raimi et al. [48] on a Nigerian population concluded that the TyG index and its anthropometric variants are effective in identifying Met-S and improving the identification and prediction of Met-S. Similar results were obtained by Zhang et al. in a Chinese population [49] and Lim et al. in a Korean population [50]. A study carried out in the USA by Jialal et al. [51] concluded that the triglycerides/HDL-C ratio increased significantly in patients with Met-S and seemed to be a valid biomarker of Met-S. Similarly, Lin et al. described that insulin resistance was closely related to alterations in lipid and glucose metabolism [52]. Therefore, IR is able to predict the risk of developing DM2 thirty years before diagnosis. Likewise, in a Chinese population, Feng found that there was a direct relationship between lipid levels (triglycerides and cholesterol) and insulin resistance [53]. We have not found any article in the consulted literature that assesses the relationship between METS-IR and Met-S; therefore, we cannot compare our results with those obtained by other authors.

A Chinese study conducted by LI et al. [54] in an adult and elderly population assessed the optimal cut-off points of the TyG index for predicting Met-S with the NCEP-ATPIII and IDF criteria. The optimal cut-off points in both cases were 8.70 (similar to ours), while the Youden indices were 0.506 for ATPIII and 0.421 for IDF, results lower than ours. In that same work, the authors investigated which of three surrogate markers (lipid accumulation product (LAP), the visceral adiposity index (VAI), and the product of triglycerides and glucose (TyG)) could better detect Met-S. The results of their study found that LAP had a higher accuracy when using the IDF criteria for Met-S, while for the ATPIII criteria, both VAI and TyG detected Met-S more accurately. This could be justified because the parameter that most influences LAP is WC, which is consistent with the MetS-IDF criterion, while WC is not among the calculation factors of TyG, and VAI includes more metabolic components in its formula. Therefore, they concluded that LAP could be a better formula for predicting Met-S than VAI and TyG. Although the determinations to detect Met-S early are different, their results coincide with those of our work.

The novelty of this study is the approach to the gap associated with diagnostic procedures and diagnostic measures for insulin resistance in adults. In addition to analysing metabolic syndrome as a risk factor for insulin resistance, we also analysed how the individual components of metabolic syndrome predispose to insulin resistance.

Where we obtained that of the elements included in the diagnosis of Met-S, the one that most seemed to increase the risk of presenting IR was waist circumference. Of the three formulas used, the IDF formula was the one that required a higher waist circumference value to be able to diagnose Met-S; hence, it is the one that is most closely related to IR. Thus, waist circumference is the central essential component for determining insulin resistance and, consequently, for the early diagnosis of metabolic syndrome [54]. It is easy to measure in the doctor’s office and does not require blood samples, which has an impact on the quality and speed of the patient’s diagnosis.

## 5. Strengths and Limitations

Among the strengths of this study, we would highlight the enormous sample size (more than 418,000 workers) and the large number of insulin resistance and metabolic syndrome risk scales applied.

As limitations, we can point out that risk scales and inexact diagnostic tests were used to assess IR. Another limitation is that the work was carried out in Spain and in the working population, so we do not know whether the results can be extrapolated to other countries and to the general population.

## 6. Conclusions

The values of the different risk scales for insulin resistance show higher values in people with metabolic syndrome when applying the three criteria.

Most risk scales for insulin resistance enable the presence of metabolic syndrome to be adequately classified, finding the best ones if the IDF criteria are applied.

Of the elements included in Met-S, the one that seems to increase the risk of presenting IR the most is waist circumference; hence, the Met-S definition that is most related to IR is that of the IDF, which is the only one of the three in which a high value of waist circumference is necessary to be able to diagnose Met-S. Waist circumference can be considered the central essential component for detecting insulin resistance and, therefore, for early detection of metabolic syndrome.

## Figures and Tables

**Table 1:** Characteristics of the population.

	Women	Men	Total	
	*n* = 172,282	*n* = 246,061	*n* = 418,343	
	Mean (SD)	Mean (SD)	Mean (SD)	*p*-Value
Age	39.6 (10.8)	40.6 (11.1)	40.2 (11.0)	<0.0001
Height	161.8 (6.5)	174.6 (7.0)	169.4 (9.3)	<0.0001
Weight	66.2 (14.0)	81.4 (14.7)	75.1 (16.2)	<0.0001
BMI	25.3 (5.2)	26.7 (4.5)	26.1 (4.8)	<0.0001
Waist	74.8 (10.6)	86.2 (11.1)	81.5 (12.2)	<0.0001
SBP	117.4 (15.7)	128.2 (15.5)	123.7 (16.5)	<0.0001
DBP	72.6 (10.4)	77.8 (11.0)	75.6 (11.0)	<0.0001
Cholesterol	190.6 (35.8)	192.6 (38.9)	191.8 (37.7)	<0.0001
HDL-c	56.8 (8.7)	50.3 (8.5)	53.0 (9.1)	<0.0001
LDL-c	116.1 (34.8)	118.0 (36.7)	117.2 (35.9)	<0.0001
Triglycerides	89.1 (46.2)	123.7 (86.4)	109.5 (74.6)	<0.0001
Glycaemia	87.8 (15.1)	93.3 (21.3)	91.0 (19.2)	<0.0001
	%	%	%	*p*-value
18–29 years	20.7	18.8	19.6	<0.0001
30–39 years	29.7	27.6	28.4	
40–49 years	29.6	30.0	29.9	
50–59 years	16.8	19.7	18.5	
≥60 years	3.2	3.9	3.6	
Social class I	6.9	4.9	5.7	<0.0001
Social class II	23.4	14.9	18.4	
Social class III	69.7	80.3	75.9	
Nonsmokers	67.2	66.6	66.9	<0.0001
Smokers	32.8	33.4	33.2	

BMI, body mass index; SBP, systolic blood pressure; DBP, diastolic blood pressure; HDL-c, high-density lipoprotein cholesterol; LDL-c, low-density lipoprotein cholesterol.

**Table 2:** Mean values by sex of the different insulin resistance risk scales according to the presence or absence of metabolic syndrome determined with different criteria.

	Non-MS ATPIII	Yes MS ATPIII		Non-MS IDF	Yes MS IDF		Non-MS JIS	Yes MS JIS	
	Mean (SD)	Mean (SD)	*p*-Value	Mean (SD)	Mean (SD)	*p*-Value	Mean (SD)	Mean (SD)	*p*-Value
Men	*n* = 204,597	*n* = 41,464		*n* = 213,558	*n* = 32,503		*n* = 178,147	*n* = 67,914	
TG/HDL-c	2.1 (1.4)	4.9 (3.2)	<0.0001	2.3 (1.8)	4.5 (3.0)	<0.0001	2.0 (1.2)	4.3 (2.9)	<0.0001
TyG index	8.4 (0.5)	9.2 (0.6)	<0.0001	8.4 (0.5)	9.0 (0.6)	<0.0001	8.3 (0.5)	9.0 (0.6)	<0.0001
TyG-BMI	215.6 (36.8)	285.4 (47.3)	<0.0001	217.0 (37.3)	295.3 (45.5)	<0.0001	210.0 (33.6)	272.8 (45.9)	<0.0001
TyG-waist	704.8 (97.2)	873.8 (121.2)	<0.0001	704.5 (94.6)	922.3 (92.2)	<0.0001	690.1 (88.6)	846.5 (116.7)	<0.0001
TyG-WtHR	4.0 (0.5)	5.0 (0.7)	<0.0001	4.0 (0.5)	5.2 (0.5)	<0.0001	4.0 (0.5)	4.9 (0.6)	<0.0001
METS-IR	37.0 (6.5)	50.3 (8.9)	<0.0001	37.3 (6.6)	52.2 (8.5)	<0.0001	36.0 (5.8)	47.9 (8.5)	<0.0001
**Women**	***n* = 155,772**	***n* = 16,510**	***p*-value**	***n* = 156,169**	***n* = 16,113**	***p*-value**	***n* = 153,102**	***n* = 19,180**	***p*-value**
TG/HDL-c	1.5 (0.7)	3.0 (1.7)	<0.0001	1.5 (0.8)	2.8 (1.5)	<0.0001	1.5 (0.7)	2.9 (1.7)	<0.0001
TyG index	8.1 (0.4)	8.8 (0.5)	<0.0001	8.1 (0.4)	8.7 (0.5)	<0.0001	8.1 (0.4)	8.7 (0.5)	<0.0001
TyG-BMI	198.9 (39.6)	287.0 (52.9)	<0.0001	198.6 (39.3)	291.8 (48.9)	<0.0001	198.0 (39.3)	281.8 (51.0)	<0.0001
TyG-waist	594.5 (84.7)	773.7 (117.3)	<0.0001	592.9 (83.5)	793.8 (95.0)	<0.0001	592.6 (84.4)	764.3 (109.9)	<0.0001
TyG-WtHR	3.7 (0.5)	4.8 (0.7)	<0.0001	3.7 (0.5)	4.9 (0.6)	<0.0001	3.7 (0.5)	4.7 (0.7)	<0.0001
METS-IR	33.7 (6.8)	49.3 (9.2)	<0.0001	33.6 (6.7)	50.2 (8.4)	<0.0001	33.5 (6.8)	48.3 (8.9)	<0.0001

TG/HDL-c, triglycerides/high-density lipoprotein cholesterol; TyG index, triglycerides glucose index; BMI, body mass index; WtHR, waist to height ratio; METS-IR, Metabolic Score for Insulin resistance; MS ATPIII, Metabolic syndrome Adult Treatment Panel III; MS IDF, Metabolic Syndrome International Diabetes Federation; MS JIS, Metabolic Syndrome Joint Interim Statement.

**Table 3:** Binary logistic regression.

	MS NCEP ATPIII		MS IDF		MS JIS	
	OR (CI 95%)	*p*-Value	OR (CI 95%)	*p*-Value	OR (CI 95%)	*p*-Value
TG/HDL normal	1	<0.0001	1	<0.0001	1	<0.0001
TG/HDL high	3.21 (3.11–3.32)		1.94 (1.87–2.01)		4.10 (3.98–4.22)	
TyG index normal	1	<0.0001	1	<0.0001	1	<0.0001
TyG index high	5.68 (5.50–5.87)		2.96 (2.85–3.06)		4.33 (4.21–4.45)	
METS-IR normal	1	<0.0001	1	<0.0001	1	<0.0001
METS-IR high	16.11 (15.64–16.58)		18.41 (17.93–18.91)		16.03 (15.55–16.53)	

TG/HDL-c, triglycerides/high-density lipoprotein cholesterol; TyG index, triglycerides glucose index; METS-IR, Metabolic Score for Insulin Resistance; MS ATPIII, Metabolic Syndrome Adult Treatment Panel III; MS IDF, Metabolic Syndrome International Diabetes Federation; MS JIS, Metabolic Syndrome Joint Interim Statement.

**Table 4:** Binary logistic regression.

		ATPIII-JIS Criteria			IDF Criteria	
	TyG High	TG/HDL High	METS-IR High	TyG High	TG/HDL High	METS-IR High
	OR (95% CI)	OR (95% CI)	OR (95% CI)	OR (95% CI)	OR (95% CI)	OR (95% CI)
Waist normal	1	1	1	1	1	1
Waist high	20.07 (19.45–20.71)	9.96 (9.68–10.25)	12.33 (11.95–12.72)	15.20 (14.76–1.85)	16.82 (16.39–17.26)	25.32 (24.27–26.41)
HDL normal	1	1	1	1	1	1
HDL high	7.33 (7.12–7.54)	1.93 (1.87–2.00)	1.66 (1.60–1.72)	1.64 (1.58–1.69)	10.09 (9.81–10.38)	7.80 (7.58–8.02)
Normal tension	1	1	1	1	1	1
High tension	2.77 (2.69–2.86)	1.33 (1.30–1.36)	1.48 (1.44–1.52)	1.44 (1.40–1.48)	1.29 (1.26–1.32)	2.30 (2.23–2.37)
TG normal	1	1	1	1	1	1
TG high	4.20 (4.08–4.32)	5.06 (4.60–5.54)	10.94 (10.56–11.46)	12.15 (11.88–12.51)	14.84 (14.39–15.52)	3.44 (3.34–3.54)
Glycaemia normal	1	1	1	1	1	1
Glycaemia high	3.16 (3.06–3.25)	1.57 (1.53–1.62)	10.58 (10.52–10.65)	12.10 11.73–12.48)	1.54 (1.49–1.58)	2.63 (2.56–2.71)

TG/HDL-c, triglycerides/high-density lipoprotein cholesterol; TyG index, triglycerides glucose index; BMI, body mass index; WtHR, waist to height ratio; METS-IR, Metabolic Score for Insulin Resistance; MS ATPIII, Metabolic Syndrome Adult Treatment Panel III; MS IDF, Metabolic Syndrome International Diabetes Federation; MS JIS, Metabolic Syndrome Joint Interim Statement.

**Table 5:** Areas under the curve (ROC curve) of the different insulin resistance risk scales for predicting the presence of metabolic syndrome applying different criteria.

	MS NCEP ATPIII	MS IDF	MS JIS
	AUC (95% CI)	AUC (95% CI)	AUC (95% CI)
Women *n* = 172,282			
TG/HDL	0.853 (0.850–0.856)	0.822 (0.819–0.826)	0.844 (0.841–0.847)
TyG index	0.846 (0.842–0.849)	0.807 (0.804–0.811)	0.837 (0.834–0.840)
TyG-BMI	0.914 (0.912–0.916)	0.937 (0.936–0.939)	0.912 (0.910–0.914)
TyG-waist	0.894 (0.891–0.896)	0.952 (0.950–0.953)	0.900 (0.897–0.902)
TyG-WtHR	0.905 (0.903–0.908)	0.950 (0.949–0.951)	0.909 (0.907–0.911)
METS-IR	0.918 (0.916–0.920)	0.942 (0.941–0.944)	0.916 (0.914–0.918)
**Men *n* = 246,061**			
TG/HDL	0.875 (0.873–0.877)	0.814 (0.812–0.817)	0.840 (0.838–0.842)
TyG index	0.868 (0.866–0.870)	0.793 (0.791–0.796)	0.828 (0.826–0.830)
TyG-BMI	0.888 (0.887–0.889)	0.919 (0.918–0.921)	0.877 (0.876–0.879)
TyG-waist	0.864 (0.862–0.866)	0.960 (0.959–0.960)	0.862 (0.861–0.864)
TyG-WtHR	0.877 (0.876–0.879)	0.950 (0.950–0.951)	0.871 (0.869–0.873)
METS-IR	0.894 (0.892–0.896)	0.925 (0.924–0.927)	0.890 (0.889–0.892)

TG/HDL-c, triglycerides/high-density lipoprotein cholesterol; TyG index, triglycerides glucose index; BMI, body mass index; WtHR, waist to height ratio; METS-IR, Metabolic Score for Insulin Resistance; MS ATPIII, Metabolic Syndrome Adult Treatment Panel III; MS IDF, Metabolic Syndrome International Diabetes Federation; MS JIS, Metabolic Syndrome Joint Interim Statement.

**Table 6:** Cut-offs, sensitivity, specificity and Youden index of the different insulin resistance risk scales for predicting the presence of metabolic syndrome applying different criteria.

	MS NCEP-ATPIII	MS IDF	MS JIS
	Cut-Off-Sens-Specif-Youden	Cut-Off-Sens-Specif-Youden	Cut-Off-Sens-Specif-Youden
Women *n* = 172,282			
TG/HDL	1.83-77.0-77.0-0.540	1.77-75.6-73.8-0.494	1.78-76.1-75.7-0.518
TyG index	8.38-76.9-76.4-0.533	8.35-73.5-73.4-0.469	8.36-76.1-75.6-0.517
TyG-BMI	236.60-83.9-83.8-0.677	241.70-86.5-86.5-0.730	234.06-83.7-83.6-0.673
TyG-waist	668.00-82.6-82.5-0.651	692.64-88.9-88.8-0.777	667.28-83.6-83.6-0.672
TyG-WtHR	4.15-84.0-83.6-0.676	4.27-88.8-88.4-0.772	4.13-84.2-84.2-0.684
METS-IR	40.25-84.4-84.4-0.688	41.24-87.3-87.2-0.745	39.58-84.6-84.6-0.692
**Men *n* = 246,061**			
TG/HDL	2.81-80.6-80.5-0.609	2.71-74.9-74.9-0.498	2.43-76.5-76.2-0.527
TyG index	8.74-80.2-80.1-0.603	8.70-73.6-73.5-0.471	8.60-76.0-75.3-0.513
TyG-BMI	246.07-80.9-80.8-0.617	253.24-83.9-83.9-0.678	236.13-79.6-79.6-0.592
TyG-waist	779.61-78.2-78.1-0.563	823.44-89.7-89.6-0.793	759.38-78.6-78.6-0.572
TyG-WtHR	4.48-79.4-79.4-0.588	4.68-88.0-87.9-0.759	4.35-79.3-79.3-0.586
METS-IR	42.40-81.5-81.4-0.629	43.82-84.7-84.6-0.693	40.59-80.9-80.9-0.618

TG/HDL-c, triglycerides/high-density lipoprotein cholesterol; TyG index, triglycerides glucose index; BMI, body mass index; WtHR, waist to height ratio; METS-IR, Metabolic Score for Insulin Resistance; MS ATPIII, Metabolic Syndrome Adult Treatment Panel III; MS IDF, Metabolic Syndrome International Diabetes Federation; MS JIS, Metabolic Syndrome Joint Interim Statement.

## Data Availability

Data are not available due to ethical or privacy restrictions.
